# The effect of decompressive craniectomy on CSF pulsatility in experimental communicating hydrocephalus

**DOI:** 10.1186/1743-8454-7-S1-S35

**Published:** 2010-12-15

**Authors:** Mark E Wagshul, Shams Rashid, James P McAllister, Martin U Schuhmann

**Affiliations:** 1 Radiology, Gruss Magnetic Resonance Research Center, Albert Einstein College of Medicine, Bronx, NY 10461, USA; 2 Biomedical Engineering, Stony Brook University, Stony Brook, NY 11794, USA; 3 Pediatric Neurosurgery, University of Utah, Salt Lake City, UT 84132, USA; 4 Pediatric Neurosurgery, University of Tubingen, Tubingen, Germany

## Background

It has been well established that intracranial pulsatility, whether measured as parenchymal/intraventricular pressure or as cerebrospinal fluid (CSF) flow in the cerebral aqueduct, is often markedly elevated in patients with hydrocephalus. Attempts have been made to use this measure to predict outcome from shunt surgery, but its specificity is still questionable. The question of whether or not intracranial pulsatility plays a causative role in hydrocephalus or is merely an epiphenomenon related to intracranial compliance is similarly an unanswered question. As a first step toward answering this question, we hypothesized that aqueductal pulsatility would be reduced after increasing compliance following decompressive craniectomy.

## Materials and methods

Communicating hydrocephalus was induced by injection of kaolin into the basal cisterns in five (n = 5) adult Sprague-Dawley rats; controls received similar saline injections. Flow pulsatility was studied at two weeks post-injection by cine phase contrast MRI prior to and immediately following bilateral craniectomy. Aqueductal stroke volume before and after craniectomy were compared. To minimize effects of extended anesthesia, the craniectomy was started prior to the first imaging scan without breaking through the bone completely. Animals were then moved to the scanner, imaged for the pre-craniectomy pulsatility measurements, and returned to the bench to complete the craniectomy. A bone flap (4 x 10 mm) was taken from each side over the parietal lobe. The dura mater was left intact, although small tears in the dura were noticed in some cases. A second scan was then obtained to measure post-craniectomy pulsatility. To ensure similar physiological state for the two scans, animals were intubated throughout and ventilated on O2/isoflurane. Following the second scan, the animals were euthanized.

## Results

All animals demonstrated moderate-to-severe ventriculomegaly (volumes from 111 – 322 microliters) and aqueductal pulsatility was elevated significantly in all cases compared to expected controls levels. In 4/5 animals, pulsatility decreased following craniectomy with a large variability in the percent reduction (17- 75 percent); the fifth animal exhibited no change. The animal with the largest decrease was also the most hydrocephalic and had the largest pre-craniectomy pulsatility.

## Conclusions

These preliminary findings appear to be the first attempt to measure CSF pulsatility while manipulating intracranial compliance experimentally. The trend toward an immediate decrease in aqueductal pulsatility suggests that compliance plays an important role in CSF pulsatility. Further studies on the effects of shunting and/or CSF infusion should be performed in order to validate this mechanism.

